# Velopharyngeal insufficiency after cleft palate repair in patients with isolated Robin sequence versus isolated cleft palate: A systematic review

**DOI:** 10.1016/j.jpra.2024.07.012

**Published:** 2024-07-30

**Authors:** N.A.T. Sullivan, V. Sijtsema, N. Lachkar, E.C. Paes, C.C. Breugem, R.J.H. Logjes

**Affiliations:** 1 Amsterdam University Medical Centers, location University of Amsterdam, department of Plastic Surgery; 2 Amsterdam Reproduction & Development Research Institute; 3 University Medical Center Utrecht, department of Plastic Surgery

**Keywords:** ‘Pierre Robin syndrome’, ‘Cleft palate’, ‘Speech’, ‘Systematic review’

## Abstract

Background: Robin sequence (RS) is characterized by micrognathia, glossoptosis, and upper airway obstruction, and is often combined with a cleft palate. It is unclear whether RS negatively impacts the development of velopharyngeal incompetence (VPI) and attainable speech outcomes. This study systematically reviewed speech outcomes in patients with cleft and isolated RS (IRS) compared with only isolated cleft palate (ICP).

Methods: A literature search following the preferred reporting items for systematic reviews and meta-analyses (PRISMA) guidelines was performed using the PubMed and EMBASE databases. Articles reporting speech outcomes following primary palatoplasty in patients with IRS only or IRS versus ICP were identified. Study characteristics and methods, primary- and VPI palatoplasty, speech measurements, and post-operative complications were collected. Primary outcomes included VPI and need for speech correcting surgery (SCS). Methodological quality was appraised using the methodological index for non-randomized studies (MINORS) criteria (range: 0-16 and 0-24).

Results: Nineteen studies reported VPI event rates that varied between 14% and 88% for IRS and 0% and 62% for ICP. Five out of 8 studies (67%) comparing VPI event rates between IRS and ICP found no significant difference. SCS rates varied between 0% and 48% for IRS and 0% and 24% for ICP. Six out of 9 studies (67%) comparing SCS rates between IRS and ICP, found no significant difference. Combined VPI event rates were 36.1% for the IRS group and 26% for the ICP group, for SCS rates this was 20% for IRS and 13% for ICP.

Conclusion: Most articles found no significant difference between the VPI and SCS rates indicating that speech outcomes might be similar in patients with IRS and ICP. To better compare these groups a standardized international protocol is needed.

**Summary of findings**

This systematic review examined speech outcomes after primary palatoplasty in isolated patients with Robin sequence (IRS) and isolated cleft palate (ICP). The analysis included 19 studies, with 914 patients with IRS and 1899 patients with ICP. Key findings include:

• Velopharyngeal insufficiency (VPI) event rates in patients with IRS (36%) compared with patients with ICP (26%).
• Speech corrective surgery (SCS) rates were also higher in patients with IRS (20%) than in patients with ICP (13%).
• Despite these differences, most studies directly comparing the 2 found no statistically significant difference in VPI or SCS rates between the IRS and ICP groups, indicating speech outcomes might be similar in patients with IRS and ICP.
• There was heterogeneity in the study designs, surgical techniques, and outcome measures across the included studies.
• Genetic screening for accurate diagnosis of IRS was inconsistently performed, potentially affecting result interpretation.
• The timing of primary palatoplasty varied widely, with no clear consensus on the optimal age for surgery.
• Standardized speech assessment methods were lacking, contributing to outcome variability.

Alt-text: Unlabelled box

## Introduction

Robin sequence (RS) is a rare congenital anomaly characterized by the triad of micrognathia, glossoptosis, and varying degrees of upper airway obstruction. In approximately 90% of cases, a cleft palate (CP) is present.^1^, ^2^, ^3^, ^4^

RS encompasses a spectrum of presentations with varying degrees of complexity and clinical manifestations that directly impact speech development and outcomes. The distinction between isolated RS (IRS) and RS associated with syndromes or genetic mutations (SRS) is crucial for understanding the nuanced challenges in managing speech outcomes. RS-plus refers to a group of patients with RS and additional congenital anomalies or chromosomal defects, but without an identified associated syndrome. IRS typically presents without additional congenital anomalies beyond the RS triad, whereas SRS involves more complex genetic backgrounds and often includes other anomalies that can further complicate speech development.^5^ Incidence of RS is 1 in 8,500 to 14,000 newborns.^5^, ^6^, ^7^, ^8^, ^9^ The exact pathogenetic physiology of RS is still unknown, with the causes for IRS being unclear.^10^^,^^11^ In SRS, the pathological mechanism is defined by mutations in chromosomes and specific genes, but the causes for IRS are still unclear.^4^^,^^10^^,^^11^

It is thought that the retrognathic tongue position causes patients with RS to have a wider and U-shaped CP in patients with RS compared with the patients with isolated CP (ICP). This complicates primary palatoplasty and may influence the approach and outcomes of speech therapy.^5^^,^^12^^,^^13^

Moreover, patients with RS can present with a varying degree of pulmonary and cardiac complications due to upper airway obstructions.^5^^,^^14^^,^^15^ The heterogeneity within the RS population, including patients with RS-plus, necessitates individualized management plans to address the unique combination of airway, feeding, and speech challenges. The additional burden of pulmonary and cardiac complications in patients with RS further complicates the clinical picture, potentially affecting speech therapy outcomes owing to the intertwined nature of respiratory and speech mechanisms. Feeding difficulties and reflux are also common symptoms, resulting in reduced weight gain and possible failure to thrive.^5^^,^^14^^,^^15^ Mortality rates vary between 1% and 26% and are mainly caused by respiratory insufficiency in combination with neurological or cardiological anomalies.^5^^,^^16^

CP can cause velopharyngeal insufficiency (VPI), the inability to sufficiently close the velopharyngeal port.^17^, ^18^, ^19^, ^20^ Clinical manifestations of VPI, such as hypernasality, nasal air emission, and an inability to produce pressure consonants, affect speech development and can result in speech and language delays. Given the critical role of speech in social integration and psychological health, understanding the distinct pathophysiology and clinical implications of RS versus ICP is essential for tailoring interventions.^17^, ^18^, ^19^^,^^21^ This nuanced approach aimed to optimize speech development outcomes by recognizing that the complexity of RS may require more specialized therapeutic strategies compared to ICP.

It is important to distinguish between IRS and SRS for clinical practices, as treatment is likely to differ.^11^ Surgical intervention for CP repair is essential for resolving feeding and hearing difficulties, minimizing facial growth disturbances, and establishing a competent velopharyngeal sphincter to achieve proper speech development. Early repair of the cleft, typically performed between 6 to 12 months after birth, is recommended to stimulate adequate velopharyngeal function.^22^, ^23^, ^24^, ^25^ However, the timing of palatoplasty in patients with RS may be delayed due to upper airway and feeding difficulties, depending on the patient's response management regarding these issues. This delay in surgical intervention can have a direct effect on speech development and outcomes, as the establishment of proper velopharyngeal function is crucial for speech articulation and intelligibility.^24^^,^^26^

The persistence of VPI after primary palatoplasty and speech correction surgery (SCS) is a significant concern in the management of patients with CP.^18^^,^^20^ VPI after primary palatoplasty requires intensive follow-up and practice by a speech and language pathologist.^4^^,^^11^^,^^22^^,^^27^, ^28^, ^29^ Approximately 6% to 19% of primary palatoplasties require SCS during follow-up.^30^ Despite efforts to standardize speech assessment methods, a gold standard is still lacking, complicating the decision-making process for performing SCS, which currently relies on expert opinions and shared decision-making.^31^^,^^32^

Some studies theorize that patients with IRS have an intrinsically normal palatal development, whereas the cause of CP in patients with ICP might be intrinsically aberrant, due to multifactorial genetic and environmental factors.^33^^,^^34^ Therefore, patients with IRS might have a better velopharyngeal function after primary palatoplasty, resulting in better speech outcomes than patients with ICP.^33^^,^^34^ Contrary to this theory, other studies suggest that after primary palatoplasty of a wide U-shaped cleft, a lack of tissue and scarring might result in a short and immobile velum, causing worse speech outcomes in patients with IRS compared to patients with ICP.^35^^,^^36^

The varying degrees of airway obstruction and congenital anomalies, bring unique challenges in the treatment of patients with RS. Several studies have focused on the treatment of RS on mandibular development, airway obstruction, and feeding problems, but few studies have compared speech outcomes of patients with RS and ICP.^4^, ^35^^,^^37^^,^^38^

So far, a single systematic review by Wan et al. (2015), compared speech outcomes after primary palatoplasty in patients with IRS and ICP. They suggested a tendency toward slightly worse speech outcomes in patients with IRS, but no significant difference was found.^39^ The review included a limited number of studies, among which half of them did not clearly provide data on speech outcomes of IRS alone.^39^ Overall, Wan et al. (2015) mentioned that the included articles provided controversial and insufficient data for drawing decisive conclusions.^39^ Hence, it is still unclear if the presence of RS negatively impacts attainable speech outcomes in infants with CPs.^4^, ^35^^,^^38^, ^39^, ^40^

This study investigated whether patients with IRS have higher incidences of VPI or SCS after primary palatoplasty compared to patients with ICP, by systematically reviewing the currently available literature.

## Methods

This review was conducted following the preferred reporting items for systematic reviews and meta-analyses (PRISMA) guidelines.

On December 1, 2023, a systematic computer-based search was performed using predefined criteria. Data were obtained from PubMed and EMBASE. The search strategy, performed by 2 independent researchers, included the following terms “Pierre Robin syndrome” and “cleft palate” and is described in detail in Supplemental Data Content 1. Inclusion criteria included: Randomized controlled trial or cohort studies, clear provision of data on speech outcomes, and 3 to 6 years of age at speech assessment. Exclusion criteria included: SRS only and articulation errors only (Supplemental Data Content 2). Additional articles were obtained from the reference lists of the previously selected articles. These articles were screened for eligibility in a similar matter.

All available data were organized and analyzed in Microsoft Excel v2207 (Microsoft Corporation (2022). Microsoft Excel. Retrieved from https://office.microsoft.com/excel). Data extracted were inclusion-exclusion criteria, study objective, genetic screening for RS, timing, type and allocation of primary surgery, number of surgeons, timing of speech evaluations and speech methods, indications, and type of SCS and other outcomes mentioned in the articles. In addition, if available, data on cleft characteristics regarding CP measurements (length and width) or CP classification were added. Further data extracted included VPI, hypernasality, need for SCS, oral nasal fistula (ONF), ONF surgery and obstructive sleep apnea (OSA). Critical appraisal of the quality of the selected studies was performed using the methodological index for non-randomized studies (MINORS).^41^

As speech measurements methods are difficult to compare, the choice was made to focus on VPI and SCS as a comparison tool for speech outcomes between the different studies from different countries.

Studies were rated on VPI as a dichotomous outcome. All speech measurements, subjective and objective, that scored general presence of VPI (mild, moderate, and severe), presence of hypernasality (mild, moderate, and severe), measurements concerning borderline-incompetent to incompetent, incompetent, and highly incompetent were considered as VPI present. If several scoring systems for VPI were used, the highest score was used in this review. In case the article mentioned a gold standard, this score was used.^25^

Data were analyzed using IBM SPSS (IBM Corp. Released 2021. IBM SPSS Statistics for Windows, Version 28.0. Armonk, NY: IBM Corp). Binomial proportion confidence intervals were calculated using the Clipper–Pearson method. Comparison of the proportions was carried out using the chi-squared or Fisher's exact tests. Odds ratios were calculated and were displayed in a mean with a 95% confidence interval. A p-value < 0.05 was considered significant.

## Results

The initial search yielded in a total of 1717 articles. After removal of duplicates, 1388 articles were screened based on title and abstract. Application of inclusion and exclusion criteria resulted in 42 articles eligible for full-text retrieval, among which 19 were included. Reference list screening identified an additional 16 articles that were not found in the initial database search. After reviewing these articles, no additional articles were added through the reference. Reasons for exclusion of articles are demonstrated in Supplemental Data Content 2.

Out of the 19 included articles, 15 had a comparative design. The mean MINORS score for these studies was 16.4 (range: 11-20). The remaining 3 studies had a noncomparative design with a mean score of 9.3 (range: 7-11; Supplemental Data File 3).

As seen in Table 1, study objectives differed among the included studies. Seven studies analyzed speech outcomes in patients with RS- and ICP,^9^, ^38^, ^42^, ^43^, ^44^, ^45^ 5 studies compared speech outcomes of patients with IRS and ICP,^35^, ^46^, ^47^, ^48^, ^49^ speech outcomes between IRS and SRS were compared in 4 studies,^32^^,^^33^^,^^37^, ^43^ and finally 4 studies evaluated the speech outcomes of the IRS population only.^21^^,^^24^^,^^50^^,^^51^

**Table 1:** Baseline characteristics of all included studies.

Reference	Objective	Inclusion	Exclusion	Study group	Gen	Sur	Prim. Sur.	Allocation primary repair technique	Air	Aud	ST	Assessment of speech	Speech check ups	Indication SCS	Treatments SCS
Butterworth et al.,^49^	Relationship between patient-related factors and speech outcomes	CP±L with speech assessment at age 5	U	ICP 1345 RS 281	U	U	U	U	U	U	Y	SLP, VPI determined with CAPS-A, hypernasality, nasal airflow	5 y	U	U
Chorney et al., 2017	Rates VPI and ONF surgery in RS and ICP	All children <18 y with CP +- CL +- submucous cleft and palatoplasty	Prior palatoplasty by other surgeon or combined procedures or PFI	iRS, 109 sRS, 27 ICP, 176	0	1	MF, 287; SL, 23; G/A, 27	All MF. SL if preoperative airway concerns or diminished need functional velum	U	U	U	‡ 1 SLP, VPI determined by hypernasality, nasal regurgitation, video fluoroscopic examination, or flexible nasopharyngoscopy	An	Clinically significant VPI or ONF based on hypernasality, nasal regurgitation, video fluoroscopic examination, nasopharyngoscopy	Pharyngeal flap iRS, 9(109); ICP, 4(176); sRS, 3(27)
de Buys Roessingh et al.,^37^	Speech outcomes in iRS vs. sRS,	RS with CP velum and hard palate in varying degree and palatoplasty	U	iRS, 25 sRS, 13	U	1	U, 38	U	PEP* PP Or-I	Y	Y	‡ 2 SLP, separate evaluations compared, VPI determined by Borel–Maisonnay: hyper/hyponasality, audible nasal emission with nasometer, voice quality, articulation errors. Score 1/2 = referred for speech therapy	An/bian	Hypernasality, weak pressure consonants, weak pharyngeal musculature, and nasal emission (type 2, 2/3, 3 Borel–Maisonny).	Pharyngeal flap iRS, 9(25); sRS, 3(13)
Evans et al.,^42^	Predictors of success after F Veau I & II in RS and ICP	All children with CP and Veau I & II and palatoplasty	Veau III & IV and unable to complete PSA	iRS, 59 sRS, 50 ICP, 162	1	2	F, 271	All F	U	N	Y	SLP, VPI determined by CAPS-A-AM, cleft-related/non-cleft-related speech errors, postoperative speech therapy. Minimal VPI = none	±3 y	U	U, iRS, 2(38); ICP, 7(93); sRS, 4(38)
Filip et al.,^9^	Multidisciplinary perspective RS and ICP	RS with CP and palatoplasty	Missing address information, decline participation	iRS, 93 sRS, 11 matched with ICP: Veau 872, VPI 351	1	U	MVL, 38; Som, 66	U	PP CPAP Na-I Or-I Trach	U	U	Different SLP, VPI determined by hypernasality, audible nasal emission, and/or velar turbulence registered as present or absent, active or passive speech disorders	U	U	Pharyngeal flap, 33(104); sphincteroplasty, 2(104); PFI, 1(104)
Goudy et al.,^35^	Rates SCS in iRS vs. ICP	iRS with CP, ≥8 y at time study, 1 formal speech assessment and palatoplasty	Syndromes	iRS, 21 matched with ICP, 42	1	1	3 flap technique, 63	All 3-flap technique	U	U	Y	‡ SLP, VPI determined by hyper/hyponasality, in case of VPI video nasal endoscopy, articulation errors, resonance	Bian	Marginal and incompetent VPI, only recommended if sufficient articulation and primary resonance disorder existed	Pharyngeal flap iRS, 2(21); ICP 9(42); double-opposing Z-plasty iRS, 1(21); ICP, 1(42)
Gustafsson et al.,^21^	Rates SCS in iRS	iRS with CP and 1-stage palatoplasty	2-stage palatoplasty, syndromes, cognitive disabilities	iRS, 78	U	5	VWK, 3; Bardach 2-flap, 45; VL, 21; Mendoza, 9	Wider clefts: VWK, overtime replaced with Bardach 2-flap. Narrower/tensionless clefts: VL or Mendoza	Or-I Na-I Trach	U	U	‡ SLP, VPI determined by nasal air emission, articulation errors, both perceptual and with nasometer, video fluorography, sometimes nasopharyngoscopy	3,5-6,8,10 >16 y	Severe VPI	Before 2005: pharyngeal flap: Honigs, 9(78); Hogan's, 2(78); After 2005 F re-palatoplasty, 23(78)
Hardwicke et al., 2015	Speechoutcomes and SCS RS vs. ICP	RS with CP and need for airway support and ICP, palatoplasty	No airway support, no 5-year follow up, syndromic ICP	iRS 21 sRS 3 ICP 24	0	3	VL±IVV, 48 Two-stage, 5	All bipedicled von langebeck, when not possible to close both hard and soft palate, also intravelar veloplasty. Reason for two-stage not mentioned.	Na-I	Y	Y	Several SLP, VPI determined by CAPS-A. cleft-related/non-cleft-related speech errors,	±65 m	U	F RS 7(24) F + buccinator flap RS 1(24) Bilateral BF RS 1(24) Hynes pharyngoplasty RS 1(24) F ICP 1(24) BF + orticochea pharyngoplasty ICP 1(24)
Kosyk et al.,^51^	Speech outcomes MDO vs. non-MDO in RS	RS with CP with Veau I or II	Genetic diagnosis of syndrome, MDO after 1 y of age	RS 158	0	U	Furlow 23 V-Y pushback 8	U	U	U	Y	VPI determined by PWSS, nasal emission, nasality score, phonation	4.5 y and 7.1 y	U	Sphincter pharyngoplasty 5 Posterior pharyngeal flap 3 Buccal myomucosal flaps 2
Logjes et al.,^44^	Surgical and speech outcomes RS vs. ICP	RS with CP and ICP and palatoplasty	Submucous CP, <4 y at PSA, lost to follow up	iRS, 34 sRS, 41 ICP, 83	1	2	SLIV, F, combined, 158	Severe and wide clefts: SLIV Mild and narrow clefts: F	TLA Trach	U	U	2 SLP, VPI determined by hyper/hyponasality, NAE/T and MCA-errors, voice disorder, all present or absent	≥4 y	Confirmed VPI	Secondary F, iRS, 3(19); ICP, 4(47); sRS, 13(25)
Logjes et al.,^43^	Surgical and speech outcomes RS vs ICP	RS with CP and ICP and palatoplasty	Severe mental retardation, no multidisciplinary assessment, no speech assessment 3-6 y, incomplete phonology submucous CP	iRS 18 sRS 23 ICP 61	1	3	VL+IVV 102	U	TLA Prone	U	U	VPI determined by hypernasality or articulation errors during assessment	4.5 y	U	Pharyngeal flap 35, redo CP repair 1
Morice et al.,^32^	Speech outcomes iRS vs. sRS	RS with CP +- airway obstruction and palatoplasty	Previous treatment elsewhere, lost to follow up, absence phonological evaluation	iRS, 96 sRS, 34	1	3	Som+ IVV, 130	Partial cleft and narrow complete cleft: one stage Wide complete cleft: first stage IVV, second stage hard palate closure	PP Na-I Or-I CPAP Trach	U	U	‡ SLP, VPI determined by Borel–Maisonnay: hyper/hyponasality, audible nasal emission, EMG testing of soft palate, muscle atrophy	U	U	Pharyngeal flap, iRS, 22(96); sRS, 11(34); secondary IVV, 2(130); PFI, 3(130)
Naros et al.,^46^	Speech outcomes iRS vs. ICP	iRS with CP only and ICP, 5-6 y at survey, palatoplasty <18 m, mixed OSA index >3/h, completed treatment palatal appliance iRS	Syndromes	iRS, 22 ICP, 22	1	2	Som + IVV +- VL, 44	All IVV +- VL	PEP**	U	Y	2 SLP, VPI determined by German Universal Reporting Parameters for Cleft Palate Speech: hyper/hyponasality, voice disorder, NAE/T, consonant production error. Blinded recordings	U	U	U, 0(44)
Palaska et al.,^50^	Treatment outcomes in iRS	iRS with CP and palatoplasty	Syndromes, incomplete case notes, lost to follow up	iRS 117	0	U	U, 117	U	PP Na-I Trach MDO	Y	Y	U	U	U	Pharyngeal flap iRS, 26(117)
Patel et al.,^33^	Speech outcomes iRS vs. sRS	RS with CP and palatoplasty, ≥4 y, Veau I or II	U	iRS, 67 sRS, 29	0	1	U, 96	U	U	U	U	‡ SLP, VPI determined by Pittsburgh Weighted Values for Speech Symptoms Associated with VPI, video fluoroscopy. Competent and borderline-competent = success	An	Borderline to borderline incompetent and incompetent	Pharyngeal flap, iRS 6(67); sRS, 10(29); double-opposing Z-palatoplasty, iRS, 3(67); sRS 3(29)
Prado-Oliveira et al.,^24^	Speech outcomes iRS F vs. VL	iRS with CP and palatoplasty with speech recordings and 1 live assessment.	U	iRS, 69	1	1	F, 33; VL, 36	U	U	U	U	‡ 1 SLP, both recordings (69 iRS + 14 random samples) and live ratings, VPI determined by 4-point scale, cul-de-sac, listeners ratings (3 listeners), nasalance with nasometer, nasal turbulence	U	NA	NA
Stransky et al.,^48^	Speech outcomes iRS vs. ICP	iRS with CP and ICP and palatoplasty, Veau I or II, clefts of the soft or soft and hard palate only.	Syndromes, hearing loss, <5 y at most recent PSA, other method than F	iRS, 55 ICP, 129	0	6	MF, 184	All MF	PP TLA Trach	U	U	2 SLP, VPI determined by Pittsburgh Weighted Values for Speech Symptoms Associated with VPI: nasality (resonance), nasal emission, facial grimace, phonation, and articulation.	U	U	U
Taku et al.,^46^	Surgical outcomes in iRS vs. ICP	iRS with CP and ICP and palatoplasty, ≥6 y at evaluation, regular speech assessments	Syndromes, submucosal cleft palate, soft palate paralysis, palatoplasty >24 m, missing data, no regular PSA	iRS, 15 ICP, 40	1	3	Pushback + F + IVV, 55	All Pushback + F + IVV	PP Or-I In-Sur	U	Y	1 SLP, VPI determined by Examination of Cleft Palate Speech Japan: hypernasality, nasal emission, nasopharyngoscopy Hypernasality + nasal emission= VPI	An	U	Double-opposing Z-palatoplasty or pharyngeal flap, combined 6(55)
Witt et al.,^38^	Rates VPI in RS vs. ICP	RS with CP and ICP and palatoplasty, feeding difficulties, overt perioperative/ postoperative airway disfunction, ≥3 y, sufficient follow up	Submucous cleft, palatoplasty elsewhere, primary pharyngeal flap, insufficient follow up, inability for full speech analysis	iRS, 34 sRS, 24 ICP, 113	U	4	Two flap + IVV, 185	All 2 flap + IVV	U	U	Y	‡ Several SLP, VPI determined by hypernasality, nasal emission, facial grimacing, compensatory misarticulations, naso endoscopy	U	Patient health, airway status, endoscopic and fluoroscopic velopharyngeal visualizations. Anatomic and functional aspects vs. morbidity and outcome.	Pharyngeal flap, sphincter pharyngoplasty, PFI

U = unclear, +- = with or without, CP = cleft palate, CL = cleft lip, PSA = perceptual speech analysis, iRS = isolated RS, sRS = syndromic or genetic mutation RS, ICP = isolated cleft palate, Gen = genetic screening, Sur = Number of surgeons, Prim. Sur = primary surgery technique, VL = von Langebeck, MVL = Modified von Langebeck, F = Furlow, MF = Modified Furlow, SL = straight-line closure, SLIV = Straight-line with intravelar veloplasty, IVV = intravelar veloplasty, G/A= gingivoperiosteoplasty/alveoloplasty, Som= Sommerlad, VWK= Veau-Wardill–Killner, PEP= pre-epiglottic plate, * = all children were fitted with a removable palatal appliance 1 week after birth, ** = all children were fitted with a Tübingen/pre-epiglottic baton palatal plate after birth, PP = prone positioning, Or-I = oropharyngeal intubation, Na-I = nasopharyngeal intubation, CPAP = continued positive airway pressure, Trach = tracheostomy, TLA = tongue-lip adhesion, MDO = mandibular distraction osteogenesis, In-Surg = invasive surgery, technique unclear, Aud = audiology check, ST = speech therapy, ‡ = objective and subjective measurements, An = annual, Bian= biannually, SLP = speech-language pathologist, VPI = velopharyngeal insufficiency, CAPS-A-AM = Cleft Audit Protocol for Speech-Augmented Americleft Modification assessment, NAE/T = audible nasal air emission/turbulence, MCA = maladaptive compensatory articulation errors, PFI = palatoplasty fat injection, Syndromes include syndromes and genetic mutations, BF = buccinator flap, PWSS = Pittsburgh Weight Speech Scores.

A comprehensive genetic screening for diagnosis of RS was performed in 8 studies, whereas 3 studies were unclear, as not all patients received genetic screening,^21^^,^^37^^,^^38^ and 5 did not mention genetic screening.^25^, ^33^, ^48^^,^^50^ Numerous techniques for primary palatoplasties were observed and can be seen in Table 2. Nine studies provided information on neonatal airway management.^9^, ^21^, ^37^, ^44^^,^^46^^,^^47^^,^^48^^,^^50^ Two studies mentioned the use of a pre-epiglottic baton plate in the treatment of neonatal airway management,^37^, ^46^ and 3 studies provided information on hearing check-ups.^37^^,^^50^ Data on speech therapy were provided in 8 articles,^35^, ^37^, ^38^, ^42^^,^^46^^,^^47^^,^^50^ and 8 studies measured speech outcomes subjectively and objectively using a nasometer or videoflourscopic examination.^21^, ^24^, ^32^, ^33^, ^35^, ^37^, ^38^, ^45^ Indications for SCS for VPI varied and were mentioned in 7 studies.^21^, ^33^, ^35^, ^37^, ^38^, ^44^, ^45^ Finally, 12 out of the 16 articles provided information on SCS techniques, among which 7 used a pharyngeal flap technique.^24^, ^42^^,^^46^^,^^48^

**Table 2:** Characteristics of study population.

Reference	Study period	Repair techniques	N of iRS	N of ICP	Cleft classification iRS	Cleft classification ICP	Cleft meas. iRS °	Cleft meas. ICP °	Age prim repair iRS (years)	Age prim repair ICP (years)	Age SE iRS (years)	Age SE ICP (years)
Butterworth et al.,^49^	2006-2014	U	281	1345	No specific information	No specific information	U	U	U	U	≥5	≥5
Chorney et al., 2017	1994-2013	Modified Furlow, straight-line or gingivoperiostoplasty	109	176	No specific information	No specific information	U	U	U	U	U	U
de Buys Roessingh et al.,^37^	1985-1998	U	25	NA	No specific information	NA	U	NA	0.58 (±0.13)	NA	U	NA
Evans et al.,^42^	2000-2014	Furlow	59	162	Veau I, 51; Veau II, 8	Veau I, 118; Veau II, 44	U	U	1.16 (1.08-1.33) °°	1 (0.85-1.08) °°	2.56 (2.20-3.54) °°	2.71 (2.20-3.70) °°
Filip et al.,^9^	1980-2010	Modified von Langebeck or Sommerlad	93	872*	No specific information	Jensen 0, 233; Jensen 1, 372; Jensen 2, 115; Jensen 3, 89; Jensen 4, 63	U	U	U	U	U	U
Goudy et al.,^35^	Unknown	3 flap technique	21	42	No specific information	No specific information	U	U	1.18 (1.00-1.50)	1.04 (0.91-1.16)	U	U
Gustafsson et al.,^21^	1990-2009	Veau-Wardill-Killner, Bardach 2-flap, von Langebeck, Mendoza	78	NA	Jensen 0, 0; Jensen 1, 0; Jensen 2, 10; Jensen 3, 48; Jensen 4,20	NA	≤9 mm, 19; 10-12 Fmm, 27; ≥13, 12	NA	0.83 (0.50-1.33) °°	NA	U	NA
Hardwicke et al., 2015	2001-2008	Von Langebeck with/without intravelar veloplasty, Some two-stage repair	21	24	No specific information	LAHSAL s, 1; S,0; hS,10; HS, 13	U	U	0.66	0.94	U	U
Kosyk et al.^51^	2000-2017	Furlow or V-Y pushback	158	NA	Veau I 42 Veau II 116	NA	U	NA	0.87 (0.79-1.00)	NA	4.6 (4.0-5.4)	NA
Logjes et al.,^44^	1990-2016	Straight-line with intravelar veloplasty or Furlow	34	83	No specific information	Jensen 0, 0; Jensen 1, 22; Jensen 2, 17; Jensen 3, 27; Jensen 4, 16	U	Grade 1, 10 Grade 2, 37; Grade 3, 30; Grade 4, 3	1.01±0.26	0.94±0.43	≥4	≥4
Logjes et al.,^43^	1993-2014	Von Langebeck with intravelar veloplasty	18	61	U	U	U	U	0.8 (0.7-2.0)	0.8 (0.7-0.9)	3.9 (3.2-7.4)	4.3 (2.9-10.2)
Morice et al.,^32^	2000-2012	Sommerlad with intravelar veloplasty	96	NA	Partial, 46; Complete, 40	NA	Soft palate cleft: narrow, 3; medium, 45; wide, 48; hard palate cleft width: <20 mm, 27; 20-40 mm, 49; >40 mm, 20	NA	1-stage 0.5±0.08 (0.46-4.5) °° 2-stage 0.5±0.11 (0.46-10) °°	NA	U	NA
Naros et al.,^46^	2008-2013∞	Sommerlad with intravelar veloplasty with/without von Langebeck	22	22	LAHSAL s, 0; S, 5; hS, 12; HS, 5	LAHSAL s, 2; S, 14; hS, 5; HS, 1	U	U	0.98±0.23	0.59±0.18	5.88±0.61	6.27±0.63
Palaska et al.,^50^	1985-2012	U	117	NA	No specific information	NA	U	NA	1.4±0.5	NA	U	NA
Patel et al.,^33^	1980-2007	U	67	NA	Veau I, 11; Veau II, 56	NA	U	NA	0.83 (0.58-1.68)	NA	≥4	NA
Prado-Oliveira et al.,^24^	Unknown	Furlow or von Langebeck	69	NA	No specific information	NA	U	NA	Furlow 1.42 (1.0-2.92) Von Langebeck 1.67 (1.0-3.67)	NA	Furlow 7.42 (5.67-9.50) Von Langebeck 5.75 (4.08-10)	NA
Stransky et al.,^48^	1981-2006	Modified Furlow	55	129	No specific information	No specific information	U	U	1.08 (0.67-2.40)	1.00 (0.25-9.00)	8.9 (5.3-11.8)	8.9 (5-18.5)
Taku et al.,^46^	2000-2011	Pushback and Furlow with intravelar veloplasty	15	40	Veau I, 8; Veau II, 7	Veau I, 23; Veau II, 17	U	U	1.54 (1.25-1.91)	1.40 (1.00-1.91)	≥ 3.5	≥ 3.5
Witt et al.,^38^	1978-1992	Two-flap with intravelar veloplasty	34	113	No specific information	No specific information	U	U	1.24	U	≥3	≥3
Total			894	1639*	**	**	**	**	**	**	**	**

N = number, Cleft meas = cleft measurements, SE = speech evaluation, U = unclear, no specific information available, NA (not applicable) = information not part of article subject, ∞ = Naros et al., 2022 prospective observational study design * Filip et al., 2015 only ICP regarding Veau, ** Not possible to calculate data, °° = Median, ° = Cleft measurement: grade 1: narrow (<5 mm), grade 2: medium (≥5 and <10 mm) grade 3: wide (≥10 and ≤14 mm) grade 4: extremely wide (≥15 mm, Veau: I: soft palate only II: hard palate + soft palate, III: soft palate to alveolus involving lip, IV: complete bilateral cleft, Jensen: 0: submucous, 1: soft palate, 2: <1/3 hard palate, 3: > 1/3 to subtotal, 4: total cleft, ICP: isolated cleft palate; LAHSAL: classification system of lip, alveolus, hard palate, soft palata, s: isolated incomplete soft, S: isolated complete soft, hS: incomplete hard, complete soft, HS: complete hard + soft.

A total number of 914 patients with IRS (range: 15-281) and 1899 patients with ICP (range: 22-1345) were retrospectively studied between 1978 to 2023. Ten studies provided information on the cleft classification, varying between Veau, Jensen, and LAHSAL among which 4 studies showed information on IRS and ICP cleft classifications.^42^^,^^46^^,^^47^^,^^51^ Cleft measurements were displayed in 3 studies.^1^^,^^21^^,^^32^ Mean age at primary palatoplasty in the IRS group was 0.94 years (range: 0.58-1.67 years) and 0.98 years (range: 0.59-1.40 years) in ICP group. Mean age at speech evaluation in the IRS group varied between 2.6 and 8.9 years and 2.7 and 8.9 years in the ICP group (Table 2).

### Primary outcomes

#### VPI event rates

**Figure 1:**
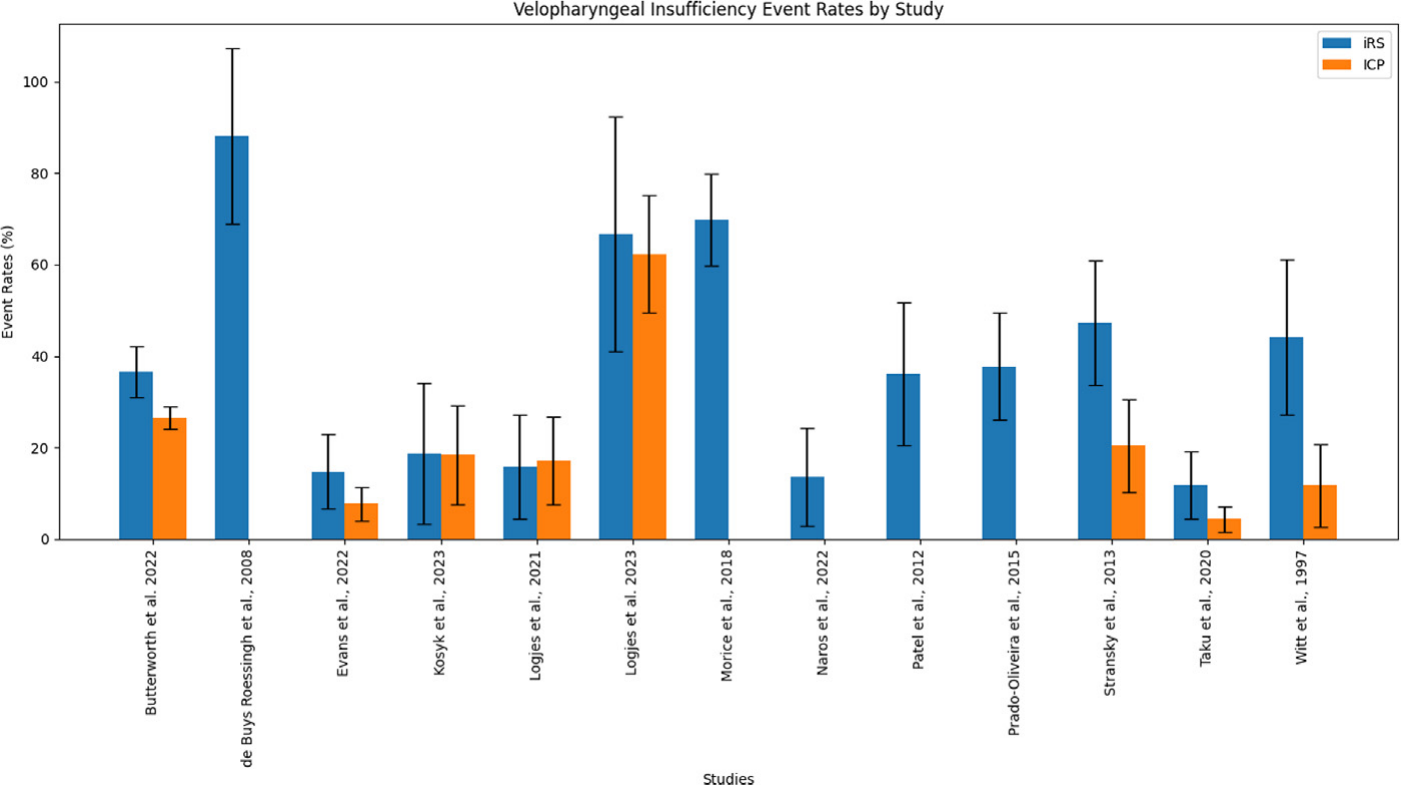
Velopharyngeal incompetence event rates, presented with 95% confidence intervals.

Five out of 8 studies (66.7%) comparing VPI event rates between IRS and ICP found no significant difference.^42^, ^43^, ^44^, ^46^, ^47^ Calculations on VPI event rates regarding IRS were performed on 10 studies (Table 3 and Figure 1). A total of 308 out of 914 patients with IRS showed signs of VPI varying from 13.6% to 69.8%. Six studies examined VPI event rates concerning IRS versus ICP. VPI was present in 461 out of 1899 patients with ICP and varied between 0% and 62.3%. Combined VPI event rates were 36.1% (33.0%-39.3%) in the IRS group versus 25.6% (23.6%-27.6%) in the ICP group.

**Table 3:** VPI event rates.

Reference	Speech correcting surgery rates						X^2^/ Fisher's exact	
	iRS		95% CI	ICP		95% CI	art p	calc p
Chorney et al., 2017	9(109)	8.3%	3.8%-15.1%	4(176)	2.3%	0.6%-5.7%	0.066	0.036
de Buys Roessingh et al.,^37^	9(25)	36.0%	18.0%-57.5%	-	-	-	-	-
Evans et al.,^42^	2(38)	5.3%	0.6%-17.7%	7(93)	7.5%	3.1%-14.9%	1.000	1.000
Filip et al.,^9^	31(93)	33.3%	23.9%-43.9%	68(351)	19.4%	15.4%-23.9%	0.004	0.007
Goudy et al.,^35^	3(21)	14.3%	3.0%-36.3%	10(42)	23.8%	12.1%-39.5%	>0.05	0.515
Gustafsson et al.,^21^	34(78)	43.6%	32.4%-55.3%	-	-	-	-	-
Hardwicke et al., 2015	10(21)	47.6%	25.7%-70.2%	2(24)	8.3%	1.0%-27.0%	0.004	0.006
Kosyk et al., 2022	9(158)	5.7%	2.6%-10.5%	-	-	-	-	-
Logjes et al.,^44^	3(19)	15.8%	3.4%-39.6%	4(47)	8.5%	2.4%-20.4%	0.401	0.401
Logjes et al.,^43^	6(18)	33.3%	13.3%-59.0%	14(61)	23.0%	13.2%-35.5%	0.37	0.373
Morice et al.,^32^	22(96)	22.9%	14.6%-32.0%	-	-	-	-	-
Naros et al.,^46^	0(22)	0.0%	-	0(22)	0.0%	-	-	-
Palaska et al.,^50^	26(117)	22.2%	15.1%-30.8%	-	-	-	-	-
Patel et al.,^33^	9(67)	13.4%	6.3%-24.0%	-	-	-	-	-
Stransky et al.,^48^	11(55)	20.0%	10.0%-31.9%	16(129)	12.4%	7.3%-19.4%	0.270	0.254
Taku et al.,^46^	3(15)	20.0%	4.3%-48.1%	3(40)	7.5%	1.6%-20.4%	0.329	0.329
Total	187(952)	19.6%	17.2%-22.3%	128(985)	13.0%	11.0%-15.3%	-	

U = unclear, art p = p-value stated in article, calc p = calculated p-value, * = significant difference with p<0.05. As none of the articles matched the experimental and control groups, and there was too much heterogeneity, it was not possible to statistically compare the total sum of the event rates.

art p = p-value stated in article, calc p = calculated p-value, * = significant difference with p<0.05. As none of the articles matched the experimental and control groups, and there was too much heterogeneity, it was not possible to statistically compare the total sum of the event rates.

#### SCS event rates

Six out of 9 studies (66.7%) comparing SCS rates between IRS and ICP groups, found no significant difference.^35^, ^42^, ^44^, ^47^, ^48^ As shown in Table 4, 16 studies allowed calculations for patients with IRS, resulting in a total of 187 out of 952 patients with IRS having SCS for VPI. Event rates varied between 0% and 47.6% among studies, with an combined effect rate of 19.6% (17.2%-22.3%). In 9 studies presenting data on SCS in patients with ICP (when compared with patients with IRS), rates varied between 0.0% and 23.8%, with a total of 128 out of 985 patients with ICP. The combined effect was 13.0% (11.0%-15.3%).

**Table 4:** SCS rates.

Reference	Velopharyngeal insufficiency event rates						χ^44^ / Fisher's exact	
	iRS		95% CI	ICP		95% CI	art p	calc p
Butterworth et al.^49^	103(281)	36.6%	31.0%-42.6%	356 (1345)	26.5%	24.1%-28.9%	0.001	0.001
de Buys Roessingh et al.,^37^	22(25)	88.0%	68.8%-97.5%	-	-	-	-	-
Evans et al.,^42^	8(54)	14.8%	6.6%-27.1%	12(156)	7.7%	4.0%-13.1%	0.170	0.175
Kosyk et al.,^51^	29(158)	18.4%	12.7%-25.3%	-	-	-	-	-
Logjes et al.,^44^	3(19)	15.8%	3.4%-36.9%	8(47)	17.2%	7.6%-30.8%	1.000	1.000
Logjes et al.^43^	12(18)	66.7%	41.0%-86-7%	38(61)	62.3%	4.9%-74.4%	0.74	0.735
Morice et al.,^32^	67(96)	69.8%	59.6%-78.7%	-	-	-	-	-
Naros et al.,^46^	3(22)	13.6%	2.9%-34.9%	0(22)	0.0%	-	>0.05	0.233
Patel et al.,^33^	11(67)	16.4%	8.5%-27.5%	-	-	-	-	-
Prado-Oliveira et al.,^24^	26(69)	37.7%	26.3%-50.2%	-	-	-	-	-
Stransky et al.,^48^	26(55)	47.3%	33.7%-61.2%	36(129)	27.9%	20.4%-36.5%	U	0.017*
Taku et al.,^46^	5(15)	33.3%	11.8%-61.6%	5(40)	12.5%	4.2%-26.8%	0.115	0.115
Witt et al.,^38^	15(34)	44.1%	27.2%-62.1%	20(113)	17.7%	11.2%-26.0%	U	0.003*
Total	330(914)	36.1%	33.0%-39.3%	486(1899)	25.6%	23.6%-27.6%	-	

art p = p-value stated in article, calc p = calculated p-value, * = significant difference with p<0.05. As none of the articles matched the experimental and control groups, and there was too much heterogeneity, it was not possible to statistically compare the total sum of the event rates.

Finally, data on OSA were provided in 4 studies,^33^^,^^37^^,^^44^, ^50^ and 1 study mentioned respiratory distress.^44^ See Supplemental Data Content 4 for more details on OSA.

## Discussion

Patients with CP are at risk for developing VPI, possibly negatively impacting a child's speech development, and causing a serious burden on the patient and their family.^17^, ^18^, ^19^^,^^21^, ^35^ To date, the impact of RS on speech prognosis concerning VPI remains unclear.^4^ Few articles have been published comparing speech outcomes in patients with IRS and ICP. This study was designed to systematically review current literature on speech outcomes after primary palatoplasty in patients with IRS and ICP and to investigate whether the presence of RS negatively impacts speech outcomes regarding VPI. The combined event rates of VPI and SCS were higher among patients with IRS than in patients with ICP. However, most articles found no significant difference between the 2 groups.

Wan et al. (2015) systematically reviewed the literature and found no significant differences between patients with IRS and ICP regarding speech outcomes.^39^ It is unclear whether only data on patients with IRS were included in this review. They mentioned 5 studies providing data on VPI, among which 3 were excluded from this study owing to unclear data on speech outcomes.^25^^,^^36^^,^^52^ Furthermore, they did not appear to make a clear distinction between IRS and SRS. Moreover, study selection and data collection were not blinded between the 2 reviewers, possibly creating a selection bias. The present review highlights an extensive heterogeneity in clinical setting and calculated outcomes, similar to Wan et al (2015), making the interpretation of compared events rates difficult to interpret.

The included studies showed mixed results regarding VPI and SCS rates. Only 3 studies found a significant difference in VPI event rates between IRS and ICP.^38^^,^^48^, ^49^ Furthermore, 2 studies described a significant difference in SCS rate, with a range of event rates from 2.3% to 19.4%.^9^^,^^40^ Interestingly, Chorney et al. 2016, did not describe a significant difference; however, using their data we found a significant difference.^45^ SCS technique differed in the studies, pharyngeal flaps and Furlow's Z-plasty were the most common used interventions. SCS is considered necessary in 6% to 19% of patients with CP and must be deliberately chosen by considering cleft characteristics and the patient's physical and psychological wellbeing.^30^, ^35^^,^^37^ Furthermore, the decision to proceed with SCS varies greatly depending on several factors, including the patient's condition, clinician's assessment, speech assessment, and surgeon's expertise. Consequently, it may not be a completely reliable outcome measure for evaluating VPI.

Although 2 studies showed a significant difference in VPI development based on cleft width and classification,^44^^,^^53^ others found no significant difference.^9^^,^^21^^,^^32^^,^^36^ There is no established gold standard for primary palatoplasty. Various surgical techniques such as Z-plasties and various different straight-line techniques have been used. It is often unknown whether radical dissection was performed or if a microscope was used during the palatoplasty. Straight-line techniques are associated with midline scarring and do not lengthen the soft palate.^54^ Interestingly, there was no significant difference in VPI outcomes between the techniques. Overall, a preferred technique for improving speech outcomes in patients with CP could not be selected based on the available data. Moreover, the lack of stratification for cleft severity in several of the reviewed studies constitutes a major confounding factor that could significantly skew the results and interpretations of the study.

Various methods were employed to assess speech, yet not every study disclosed its specific approach for speech evaluation.^9^, ^35^^,^^50^ The utilization of objective measures for VPI, such as videofluoroscopy or nasopharyngoscopy, is notably underreported. Although it is often recommended to assess speech before the age of 5 years, this guideline was not universally followed across the studies.^24^, ^48^ The timing and frequency of evaluations are crucial, as the outcomes of speech testing can be affected by factors such as the patient's motivation, fatigue, and psychological state. Although speech therapy was implemented in 7 studies,^9^, ^35^, ^37^, ^38^, ^42^, ^46^, ^47^, ^50^ its precise impact remains unclear and warrants further investigation. The absence of standardized speech testing methods likely contributes to, and may indeed be the primary cause of, the significant heterogeneity observed among the included articles.

The timing of primary palatoplasty exhibits considerable variation across studies, underscoring the ongoing debate regarding the most advantageous period for CP closure. Although earlier surgical intervention is generally linked to enhanced speech outcomes, the formation of surgical scar tissue following CP repair can potentially impede maxillary growth, leading to craniofacial anomalies.^55^ Furthermore, a significant body of research has explored the efficacy of a two-stage repair approach. However, this strategy has been implicated in less favorable long-term speech outcomes.^32^^,^^40^ The substantial heterogeneity observed in speech outcomes across all included articles in the study indicates that the relationship between surgical timing, technique, and speech development outcomes remains complex and multifaceted, necessitating further investigation to establish optimal treatment protocols.

The accurate diagnosis and differentiation IRS and SRS are critical for the assessment and treatment of VPI, as the etiology and management may differ significantly between the 2 conditions. In the current literature, there is a lack of consensus regarding the impact of syndromes on the incidence of VPI, with some studies reporting no significant difference between IRS and SRS, whereas others have observed notable disparities.^11^ This inconsistency may be attributed, in part, to the absence of genetic screening in 10 of the studies reviewed,^9^, ^21^, ^33^, ^35^, ^37^, ^38^, ^40^, ^48^, ^50^ which could lead to the inadvertent inclusion of undiagnosed syndromic cases within the IRS group. The omission of genetic screening hinders the precise categorization of patients, potentially conflating IRS with SRS and contributing to the observed heterogeneity in VPI event rates.^49^^,^^56^ However, the influence of syndromes on VPI remains unclear, with some studies finding no significant difference in VPI incidence between IRS and SRS,^37^, ^42^, ^44^, ^45^, ^46^ while others finding a significant difference.^32^^,^^33^ Genetic screening plays a pivotal role in accurately distinguishing between IRS and SRS, ensuring that patients are correctly classified, which is essential for understanding the true incidence of VPI and tailoring appropriate treatment strategies. Therefore, incorporating genetic screening into the diagnostic process is indispensable for advancing our comprehension of VPI in the context of RS and for the development of more effective clinical protocols.

Regarding OSA, there is insufficient data to make conclusive statements.^9^^,^^45^ Previous literature has outlined various perioperative protocols, including the use of standard polysomnography, to monitor these patients effectively.^16^, ^27^, ^44^^,^^57^^,^^58^ This gap in accurate ONF reporting underscores the need for more rigorous and transparent documentation of surgical outcomes to better understand and mitigate the risks associated with CP repair.

The current review has several strengths, including being the largest series of studies that provide data on VPI and SCS rates in individuals with IRS. The study methodology and patient characteristics are presented in detail, facilitating comparisons. However, there are limitations to be acknowledged. The broad search strategy aimed to minimize selection bias but resulted in heterogeneity. All but one study had a retrospective design, possibly leading to selection bias. Although hearing outcomes are an important outcome in relation to speech outcomes, this was not within the scope of our review; therefore, we did not review this subject. We only included studies involving patients with ICP if they also included patients with RS, possibly leading to underreporting and observer bias. Non-standardized operating techniques created a bias, possibly impacting speech outcomes. Relevant data, such as cleft characteristics and VPI severity, were incompletely reported. Finally, the study did not focus on the outcomes of SCS.

This systematic review has uncovered a significant degree of heterogeneity in the reported speech outcomes among patients with IRS, which has precluded the effective use of traditional meta-analysis and multivariate analysis techniques. Despite this variability, the largest studies included in the review identified significant differences in VPI rates between the patients with IRS and ICP, suggesting that there may indeed be a distinction in speech outcomes between these groups. However, to draw definitive conclusions, further research is imperative. The establishment of an international, multidisciplinary protocol for CP management is essential to enhance the consistency and quality of cleft care globally. Such a protocol should encompass standardized approaches to palatoplasty, SCS, genetic screening, cleft measurements, and speech testing. Future investigations should delve into the influence of speech therapy, SCS, airway management, and hearing status on speech outcomes. These studies should employ multicenter prospective designs with blinded speech assessments and an intention-to-treat analysis framework. Additionally, research efforts should be directed toward elucidating the pathophysiological differences between CP and RS to better understand their impact on VPI and associated speech outcomes.

## Conclusions

**Figure 2:**
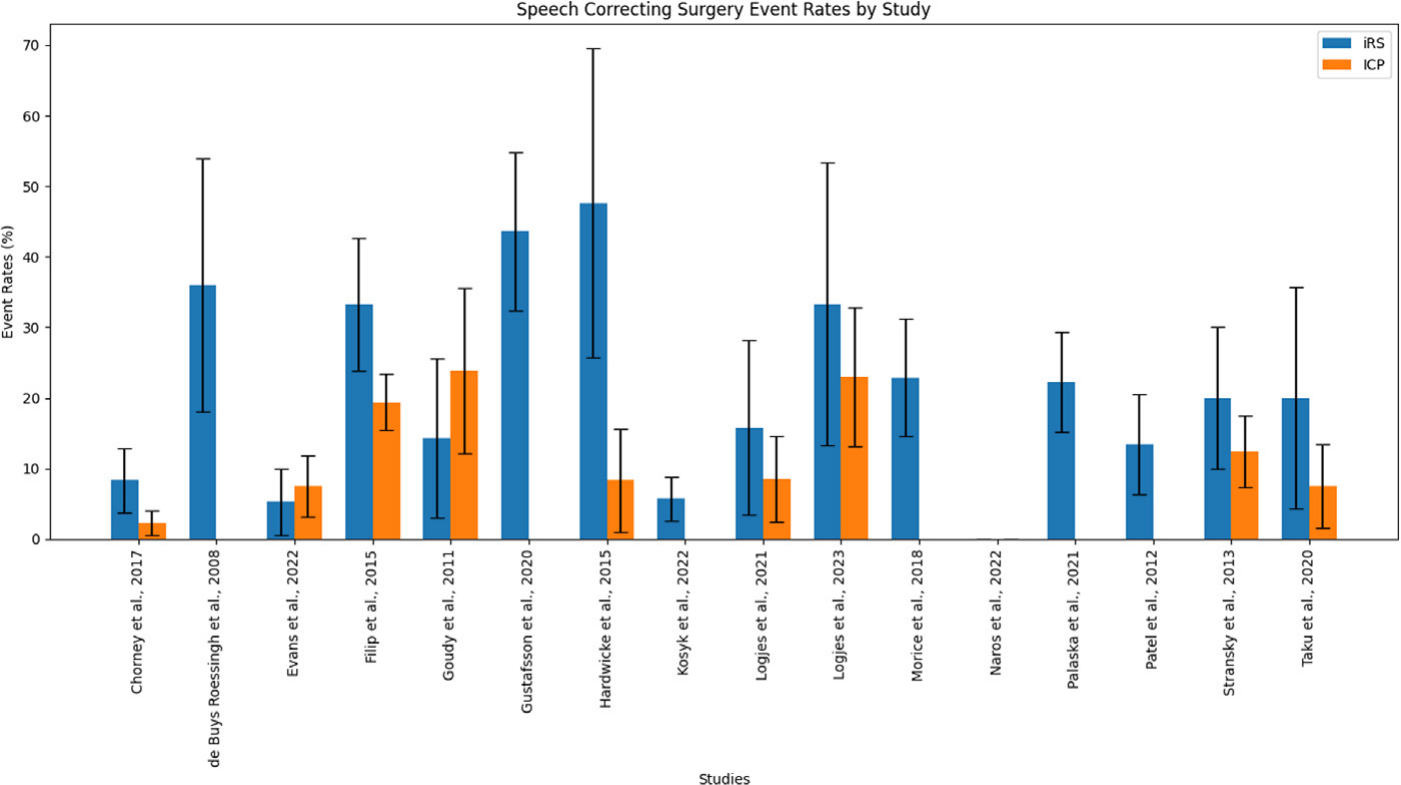
Speech correcting surgery event rates, presented with 95% confidence intervals.

The majority of the included articles did not report a significant difference in speech outcomes between patients with IRS and ICP, suggesting that speech outcomes in patients with IRS may be comparable with those in patients with ICP. To enhance the consistency of future research, the implementation of an international standard protocol for CP treatment and assessment is essential. Figure 2.

## Appendix Supplementary materials

Image, application 1

Image, application 2

Image, application 3

Image, application 4
