# Supplementary material for: Velopharyngeal insufficiency after cleft palate repair in patients with isolated Robin sequence versus isolated cleft palate: A systematic review
Source: JPRAS Open. 2024 Jul 30;42:58–80. doi: 10.1016/j.jpra.2024.07.012 (PMC11405636; doi:10.1016/j.jpra.2024.07.012)
Supplement: Supplementary file 1 [file mmc1.docx]

# Supplementary Digital Content 1: Search strategy

### Search PUBMED

| **Search**  **10-6-22** | **Query** | **Results** |
| --- | --- | --- |
| #3 | **#1 AND #2** | 761 |
| #2 | **"Cleft Palate"[Mesh] OR "cleft palate"[tiab] OR "orofacial cleft"[tiab] OR "palatal defect"[tiab] OR "palatoschisis"[tiab] OR "u-shaped"[tiab] OR "u shaped"[tiab]** | 38551 |
| #1 | "pierre robin syndrome"[Mesh] OR "pierre robin sequence"[tiab] OR "pierre robin syndrome" [tiab] OR "pierre-robin sequence"[tiab] OR "pierre-robin syndrome"[tiab] OR "robin syndrome" [tiab] OR "robin sequence"[tiab] OR "robin anomal*"[tiab] OR "pierre robin anomal*"[tiab] OR "PRS"[tiab] OR "RS"[tiab] OR "micrognathia glossoptosis"[tiab] OR "Pierre Robin association"[tiab] OR "robin malformation"[tiab] OR "pierre robin malformation"[tiab] OR "pierre robin malformation"[tiab] OR "pierre-robin malformation"[tiab] OR "pierre-robin anomal*"[tiab] OR "pierre-robin association"[tiab] | 59638 |

### Search EMBASE

| **Search 14-06-22** | **Query** | **Results** |
| --- | --- | --- |
| #4 | #1 AND #2 AND [embase]/lim | 998 |
| #3 | #1 AND #2 | 1242 |
| #2 | 'cleft palate'/exp OR 'cleft palate':ti,ab,kw OR 'orofacial cleft':ti,ab,kw OR 'palatal defect':ti,ab,kw OR 'palatoschisis':ti,ab,kw OR 'u-shaped':ti,ab,kw OR 'u shaped':ti,ab,kw | 45214 |
| #1 | 'pierre robin syndrome'/exp OR 'pierre robin syndrome' OR 'pierre robin sequence':ti,ab,kw OR 'pierre robin syndrome':ti,ab,kw OR 'pierre-robin sequence':ti,ab,kw OR 'pierre-robin syndrome':ti,ab,kw OR 'robin syndrome':ti,ab,kw OR 'robin sequence':ti,ab,kw OR 'robin anomal*':ti,ab,kw OR 'pierre robin anomal*':ti,ab,kw OR 'prs':ti,ab,kw OR 'rs':ti,ab,kw OR 'micrognathia glossoptosis':ti,ab,kw OR 'pierre robin association':ti,ab,kw OR 'robin malformation':ti,ab,kw OR 'pierre robin malformation':ti,ab,kw OR 'pierre-robin malformation':ti,ab,kw OR 'pierre-robin anomal*':ti,ab,kw OR 'pierre-robin association':ti,ab,kw | 78697 |

### Table, Supplementary Digital Content 1:

Search strategy
