# Supplementary material for: Velopharyngeal insufficiency after cleft palate repair in patients with isolated Robin sequence versus isolated cleft palate: A systematic review
Source: JPRAS Open. 2024 Jul 30;42:58–80. doi: 10.1016/j.jpra.2024.07.012 (PMC11405636; doi:10.1016/j.jpra.2024.07.012)
Supplement: Supplementary file 2 [file mmc2.docx]

# Supplementary Digital Content 2: PRISMA flowchart

**Identification of studies via other methods**

**Identification of studies via databases and registers**

Records identified from:

Citation searching (n =16)

Duplicates removed: 408

Records marked as ineligible by automation tools: 0

Records removed for other reasons: 0

Records identified from:

Databases: 1759

Registers: 0

**Identification**

Records excluded: 1309

*Exclusion criteria: sRS only, articulation errors only, n < 10, palatoplasty > 2 years, speech outcomes during adulthood, non-human studies, case reports, case-control series and reviews*

Records screened: 1351

*Inclusion criteria: English articles, availability of full-texts, RCT or cohort studies, clear provision of data on speech outcomes, 3-6 years of age at speech assessment*

**Screening**

Reports sought for retrieval: 42

Reports not retrieved: 2

Reports sought for retrieval: 16

Reports not retrieved due to wrong publication type: 12

Reports excluded:

No clear description of outcome/different outcome: 3

Inaccurate population: 9

No clear description of population: 1

Wrong age limit: 1

Reports assessed for eligibility: 14

Reports excluded:

No clear description of iRS data: 7

<10 iRS patients: 2

Inaccurate population: 2

Reports assessed for eligibility: 30

Studies included in review: 19

Reports of included studies: 0

**Included**

*Table, Supplementary Digital Content 2:*

PRISMA flowchart
