# Supplementary material for: Velopharyngeal insufficiency after cleft palate repair in patients with isolated Robin sequence versus isolated cleft palate: A systematic review
Source: JPRAS Open. 2024 Jul 30;42:58–80. doi: 10.1016/j.jpra.2024.07.012 (PMC11405636; doi:10.1016/j.jpra.2024.07.012)
Supplement: Supplementary file 3 [file mmc3.docx]

# Supplementary Digital Content 3: Critical appraisal using MINORS criteria

| **Reference/Question** | **1** | **2** | **3** | **4** | | **5** | **6** | **7** | | **8** | **9** | **10** | **11** | | **12** | **Total** | |
| --- | --- | --- | --- | --- | --- | --- | --- | --- | --- | --- | --- | --- | --- | --- | --- | --- | --- |
| Butterworth et al., 2022 | 2 | 2 | 1 | 2 | | 0 | 2 | 0 | | 0 | 2 | 2 | 1 | | 2 | 16(24) ▫ | |
| Chorney et al., 2017 | 2 | 2 | 2 | 1 | | 0 | 2 | 0 | | 0 | 1 | 2 | 0 | | 1 | 13(24) | |
| de Buys Roessingh et al., 2008 | 2 | 2 | 1 | 2 | | 0 | 2 | 0 | | 0 | 2 | 2 | 0 | | 1 | 14(24) | |
| Evans et al., 2022 | 1 | 2 | 2 | 1 | | 0 | 2 | 1 | | 0 | 2 | 2 | 1 | | 2 | 16(24) | |
| Filip et al., 2015 | 1 | 2 | 2 | 1 | | 0 | 1 | 0 | | 0 | 2 | 2 | 1 | | 1 | 13(24) | |
| Goudy et al., 2011 | 1 | 2 | 1 | 2 | | 0 | 1 | 0 | | 0 | 2 | 0 | 0 | | 2 | 11(24) | |
| Gustafsson et al., 2020 | 2 | 2 | 2 | 2 | | 0 | 2 | 1 | | 0 | - | - | - | | - | 11(16) ▫ | |
| Hardwicke et al., 2016 | 2 | 2 | 2 | 2 | | 0 | 2 | 1 | | 0 | 2 | 2 | 2 | | 2 | 19(24) | |
| Kosyk et al., 2023 | 2 | 2 | 2 | 2 | | 0 | 2 | 0 | | 0 | - | - | - | | - | 10(16) ▫ | |
| Logjes et al., 2021 | 2 | 2 | 2 | 2 | | 0 | 2 | 1 | | 0 | 2 | 2 | 2 | | 2 | 19(24) | |
| Logjes et al., 2023 | 2 | 2 | 2 | 2 | | 0 | 2 | 0 | | 0 | 2 | 2 | 2 | | 2 | 18(24) | |
| Morice et al., 2018 | 2 | 2 | 2 | 2 | | 0 | 2 | 1 | | 0 | 2 | 2 | 2 | | 2 | 19(24) | |
| Naros et al., 2022 | 2 | 2 | 2 | 2 | | 2 | 2 | 0 | | 0 | 2 | 2 | 2 | | 2 | 20(24) | |
| Palaska et al., 2021 | 1 | 2 | 2 | 0 | | 0 | 2 | 0 | | 0 | - | - | - | | - | 7(16) ▫ | |
| Patel et al., 2012 | 2 | 2 | 2 | 2 | | 0 | 2 | 1 | | 0 | 2 | 2 | 2 | | 1 | 18(24) | |
| Prado-Oliveira et al., 2015 | 2 | 2 | 2 | 2 | | 1 | 2 | 0 | | 0 | 2 | 2 | 0 | | 2 | 17(24) | |
| Stransky et al., 2013 | 2 | 2 | 2 | 2 | | 0 | 2 | 0 | | 0 | 2 | 2 | 1 | | 2 | 17(24) | |
| Taku et al., 2020 | 2 | 2 | 2 | 2 | | 0 | 2 | 0 | | 0 | 2 | 2 | 2 | | 1 | 17(24) | |
| Witt et al., 1997 | 2 | 2 | 2 | 1 | | 0 | 2 | 0 | | 0 | 2 | 2 | 0 | | 2 | 15(24) | |
| **Mean** | Non-comparative | | | | 9.3 (range: 7-11) | | | | Comparative | | | | | 16.4 (range: 11-20) | | |  |

*Table, Supplementary Digital Content 3:*

MINORS critical appraisal, ▫ = non-comparative study design
