# Supplementary material for: Velopharyngeal insufficiency after cleft palate repair in patients with isolated Robin sequence versus isolated cleft palate: A systematic review
Source: JPRAS Open. 2024 Jul 30;42:58–80. doi: 10.1016/j.jpra.2024.07.012 (PMC11405636; doi:10.1016/j.jpra.2024.07.012)
Supplement: Supplementary file 4 [file mmc4.docx]

# Supplementary Digital Content **4: Acute respiratory distress and OSA in follow up**

|  | **OSA RS** | **OSA iRS** | **OSA ICP** | **ARD** | **Age when OSA is diagnosed** |
| --- | --- | --- | --- | --- | --- |
| de Buys Roessingh et al., 2008 | 0(38) | - | - | - | 1 and 6 months post-operatively |
| Logjes et al., 2021 | - | 2(19) | 1(47) | 0 | RS 4.8 years (2.9–6.3) °°  ICP 10.3 years |
| Palaska et al., 2021 | - | 21(117) | - | - | U |
| Patel et al., 2012 | 0(96) | - | - | - | U |

*Table, Supplementary Digital Content 5:*

Acute respiratory distress and OSA in follow up, RS = Robin sequence, iRS = isolated Robin sequence, ICP = isolated cleft palate, U = unclear, °° = median, OSA = obstructive sleep apnea, ARD = acute respiratory distress.
